# Remedy for La Grippe

**Published:** 1890-11

**Authors:** 


### Remedy for La Grippe.

We feel wedded, not to la grippe, but to the remedies mentioned for its relief. We believe the sheet-anchor of treatment to be the following formula:

Acetanilid.................................. ......one drachm.
Tongaline.........................................three ounces.
Elixir of lactopeptine.........................three ounces.

A dessert spoonful every two to four hours is indicated, accompanied in some cases by the carbonate of soda. Convalescence is slow; the alimentary tract is more or less disturbed. More of the cases in our observation have had catarrhal disturbances of the digestive tract than of the air passages. The nervous system is particularly "rattled," and recovery of tone on its part is very slow. Tonics later are indicated.—*Med. Mirror.*

Aseptic Operations.—Fritsch (*Deutsche Med. Wochenschr.*) claims that the most important discovery in modern antisepsis is that carbolic acid and sublimate are not needed in fresh, clean wounds; that dirty wounds must be cleaned goes without saying, but clean tissues are only injured by antiseptics. Fritsch has of late used only 0.6 per cent. sterilized solution of sodium chloride, in all operations, even the severer ones in the abdominal cavity. When this cavity is irrigated with solutions of carbolic, salicylic, or boracic acids, depressed heart's action and collapse frequently appear. A directly opposite condition is produced by irrigation with warm salt solution; indeed, this method may be used to prevent the more serious symptoms of surgical collapse.

Disinfection of the Hands.—Ball (*Deutsche Med. Wochenschr.*) has studied carefully several methods, and has come to the conclusion that that recommended by Miculicz is the safest and best. It consists essentially in: 1. The removal of visible dirt from the finger-nails with knife or shears. 2. Then the hands are washed and brushed for three minutes in warm water with potash soap. 3. One-half minute in 3 per cent. carbolic acid solution, and then rinsed in a 1:2000 sublimate solution. 4. Lastly, the space under the nails and the root of the nails are rubbed with damp iodoform gauze for about one minute.

Ball has made some experiments with pure cultures of the staphylococcus areus, with which he infected his hands, and then applied this method. The result showed that it was a certain means of sterilizing the hands, it being a matter of indifference whether they were previously infected or not.

